# Rescue of NLRC5 expression restores antigen processing machinery in head and neck cancer cells lacking functional STAT1 and p53

**DOI:** 10.1007/s00262-023-03589-y

**Published:** 2024-01-17

**Authors:** Brendan L. C. Kinney, Sreenivasulu Gunti, Vikash Kansal, Connor J. Parrish, Nabil F. Saba, Yong Teng, Mary Katherine Henry, Fang-Yi Su, Gabriel A. Kwong, Nicole C. Schmitt

**Affiliations:** 1 Department of Otolaryngology – Head and Neck Surgery, Head and Neck Cancer Program, Winship Cancer Institute, Emory University School of Medicine, 550 Peachtree Street NE, 11Th Floor Otolaryngology, Atlanta, GA 30308 USA; 2 Winship Cancer Institute, Emory University, Atlanta, GA USA; 3 National Institute of Deafness and Communication Disorders, NIH, Bethesda, MD USA; 4 Saint Louis University School of Medicine, St. Louis, MO USA; 5 Department of Hematology and Medical Oncology, Emory University School of Medicine, Atlanta, GA USA; 6 Wallace H. Coulter Department of Biomedical Engineering, Georgia Institute of Technology and Emory School of Medicine, Atlanta, GA USA; 7 Emory University School of Medicine, Atlanta, GA USA

**Keywords:** STAT1, p53, Antigen processing machinery, NLRC5, Head and neck squamous cell carcinoma, Head and neck cancer

## Abstract

The antigen processing machinery (APM) components needed for a tumor cell to present an antigen to a T cell are expressed at low levels in solid tumors, constituting an important mechanism of immune escape. More than most other solid tumors, head and neck squamous cell carcinoma (HNSCC) cells tend to have low APM expression, rendering them insensitive to immune checkpoint blockade and most other forms of immunotherapy. In HNSCC, this APM deficiency is largely driven by high levels of EGFR and SHP2, leading to low expression and activation of STAT1; however, recent studies suggest that p53, which is often mutated in HNSCCs, may also play a role. In the current study, we aimed to investigate the extent to which STAT1 and p53 individually regulate APM component expression in HNSCC cells. We found that in cells lacking functional p53, APM expression could still be induced by interferon-gamma or DNA-damaging chemotherapy (cisplatin) as long as STAT1 expression remained intact; when both transcription factors were knocked down, APM component expression was abolished. When we bypassed these deficient pathways by rescuing the expression of NLRC5, APM expression was also restored. These results suggest that dual loss of functional STAT1 and p53 may render HNSCC cells incapable of processing and presenting antigens, but rescue of downstream NLRC5 expression may be an attractive strategy for restoring sensitivity to T cell-based immunotherapy.

**Supplementary Information:**

The online version contains supplementary material available at 10.1007/s00262-023-03589-y.

## Introduction

Approximately half of head and neck squamous cell carcinomas (HNSCCs) recur after standard therapy. Anti-PD-1 immune checkpoint blockade (ICB) is the recommended first-line treatment for recurrent/metastatic HNSCCs that express PD-L1. In cases where PD-L1 expression is low or rapid cytoreduction is needed, PD-1 ICB + platinum chemotherapy is recommended [1]. Most patients fail to respond, and there is an unmet clinical need for biomarkers to identify patients who will not benefit from PD-1-targeted immunotherapy or chemoimmunotherapy. Novel treatment strategies are also needed for these checkpoint-resistant patients.

**Fig. 1:**
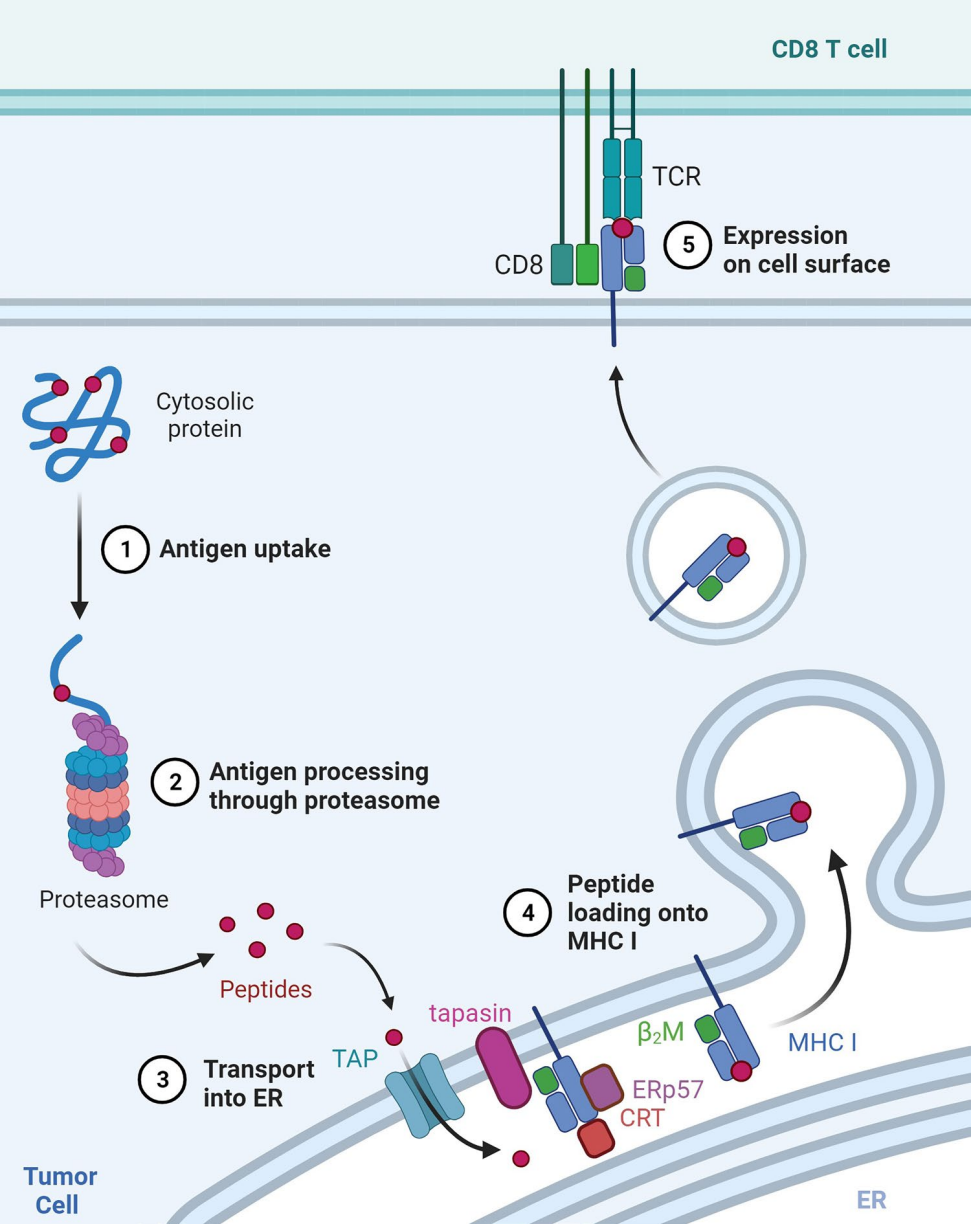
Schema of antigen processing machinery. Created with Biorender.com, with license

Several factors are required for effective anti-tumor immunity: presence of immune effector cells, recognizable tumor antigens, and intact interferon (IFN) signaling pathways, including transcription of the antigen processing machinery (APM; Fig. 1) required for processing and presentation of tumor antigens to cytotoxic T cells. Low levels of APM components such as transporter associated with antigen processing 1 (TAP1) and major histocompatibility complex (MHC) class I (also known as human leukocyte antigen or HLA in humans) are a prominent mechanism of immune escape in HNSCC and other solid tumors [2–4]. Patients whose tumors lack functional machinery for antigen processing and presentation cannot respond to currently available forms of immunotherapy, including ICB and adoptive T cell therapy [5, 6]. Increased APM component expression in response to IFN- γ is largely dependent on signal transducer and activator of transcription 1 (STAT1), which is also activated by cisplatin chemotherapy [7, 8]. Our prior work suggests that cisplatin enhances anti-tumor immunity, in part by increasing APM component expression [9, 10]. Interestingly, we noted lower APM expression in HNSCC cells lacking wild-type p53, which is also known to be important for cisplatin-induced cell death [10, 11]. Indeed, one prior study reported that physical interaction between p53 and STAT1 is required for optimal production of TAP1 [12]. Given the high incidence of *TP53* mutation in HNSCC [13], it is plausible that lack of p53 function contributes to APM deficiencies in HNSCC.

APM component deficiencies that can be rescued by exogenous interferon have been described as “soft” deficiencies, whereas “hard” deficiencies cannot be rescued with appropriate stimuli [5]. In HNSCC, high expression of epidermal growth factor receptor (EGFR) leads to high levels of Src homology-2 domain-containing phosphatase (SHP)-2, which dephosphorylates STAT1 and curtails IFN-induced upregulation of APM components [3, 4]. It has recently been shown that nod-like receptor family caspase recruiting domain-containing 5 (NLRC5) is the transcription factor downstream of STAT1 responsible for upregulation of most APM components. We hypothesized that the high incidence of *TP53* mutation and the known lack of activated STAT1 are both major contributors to APM deficits in HNSCC.

In the current study, we aimed to evaluate the depth of the APM deficit brought on by the loss of p53 and/or STAT1 and the potential for rescue with NLRC5 replacement in vitro. We hypothesized that p53 loss results in a soft APM deficiency, and the loss of both P53 and STAT1 results in a hard APM deficiency where cells are no longer able to increase APM expression regardless of stimulus. We utilized clustered regularly interspaced palindromic repeats (CRISPR)-Cas9 technology to knock out *TP53* and *STAT1* in multiple cell lines, then rescued downstream *NLRC5* expression. We also examined mRNA expression data from The Cancer Genome Atlas (TCGA).

## Methods

### Cell lines

JHU029 cells were obtained from Dr. David Sidransky and HCT116 cells from Dr. Bert Vogelstein, both at Johns Hopkins University. UM-SCC-74A cells were obtained from Dr. Thomas Carey at the University of Michigan. UPCI SCC-90 cells were purchased from ATCC. Mouse oral cancer (MOC1) cells were obtained from Kerafast. Cell lines were validated by short tandem repeat testing and/or HLA typing. For long-term storage, cells were kept in liquid nitrogen. Cells were regularly tested for Mycoplasma contamination and passaged for no more than 3 months or 20 passages before discarding.

### *CRISPR-Cas9 gene knockout and *in vitro* treatment of cell lines*

The UM-SCC-74A *TP53-/-* knock out pool was generated by Synthego (Synthego, CA, USA) as previously described [14], and then, individual clones were prepared by limiting dilution. One of these *TP53-/-* clones (clone 33), and the JHU029 cells, were then sent to Synthego for *STAT1* knockout by CRISPR-Cas9 using guide RNA 5’GGUGGCAAAUGAAACAUCAU3’. Knockout efficiency of edited pool was determined by genomic DNA sequencing using the primers forward 5’AGTGTGTGCTCAATTGTATTTGCT3’ and reverse 5’ATGAACACTGTCATGCACAATCTC3’. The *TP53-/-* clones were further validated using transcriptional assays (Supplemental Figure S1B). P53 transcriptional activity assay was performed using TransAM DNA-binding ELISA kit as per manufacturer instructions. Briefly, nuclear extracts were prepared from cells treated with cisplatin or not and incubated on plates pre-coated with oligo specific for p53. Bound p53 activity was then determined using an antibody specific for p53 by constructing a standard curve, per manufacturer instructions. The *STAT1-/-* cell pool was not taken to clones but showed > 90% knockout by flow cytometry, which was routinely repeated alongside experiments (Figure S1C). For MOC1 cells, the murine STAT1 CRISPR-Cas9 kit was obtained from Synthego and used to knock out STAT1 in parental MOC1 cells, following the manufacturer’s instructions. After verifying absence of STAT1 protein expression by immunofluorescence and flow cytometry, individual clones were prepared by limiting dilution.

To stimulate DNA damage and/or antigen processing machinery, cells were treated for 24–48 h with pharmaceutical grade cisplatin (1 µg/ml; McKesson) and/or human recombinant IFN-γ (10 ng/ml; BioLegend) and then analyzed by flow cytometry or immunofluorescence.

### Flow cytometry

After in vitro treatments, cells were harvested with trypsin/EDTA, rinsed in PBS, fixed, and then permeabilized with the eBioscience kit prior to intracellular staining. Samples were then analyzed on a BD Symphony A3 cytometer and then further analyzed using FlowJo software. Live cells were gated based on negative staining for FVS575 viability dye (BD Biosciences) or Zombie UV (BioLegend). “Fluorescence minus one” controls were tested for each multicolor flow panel. The mean fluorescence intensity from the isotype control was subtracted from each sample and then calculated as a mean fold change versus untreated wild-type cells. Antibodies and their corresponding isotype controls were from Sigma (TAP 1 unconjugated, MABF125), Abcam LMP2 AF647, Ab106824; ERp57 FITC, Ab183396; calreticulin PE, AB209577), BD Biosciences (HLA-A,B,C BUV805, B742025; STAT1 PE, 558,537), or BioLegend (PD-L1 BV650, 329,740; b2 microglobulin APC/Fire 750, 316,314; rat anti-mouse PECy7 secondary antibody for TAP1, 406,613).

### T cell killing experiments

MOC1 cells were plated in 96-well plates E-plates (ACEA Biosciences) compatible with the xCELLigence real-time cell analyzer and allowed to adhere overnight. Tumor infiltrating lymphocytes were obtained and added to a selection of culture wells on the following day, as previously described [10, 15, 16]. Briefly, MOC1 cells were inoculated subcutaneously into immunocompetent mice. After 10–14 days, tumors were harvested and minced into small pieces and then cultured with IL-2 for several days. CD8+ T cells were then magnetically sorted and counted before adding to the tumor cell culture. The electrical impedance was normalized at the time of adding T cells, and the change in impedance was compared among treatment groups.

### NLRC5 plasmid transfection, immunofluorescence, and confocal microscopy

The FLAG-NLRC5 plasmid (#37,521) [17] was obtained from Addgene and transfected into *STAT1-/-* cell lines according to the manufacturer instructions. To create stable cell lines expressing NLRC5, transfected cells were selected by G418 resistance after incubation with G418 for two passages. For detection of NLRC5, cells were cultured in chamber slides for up to 24 h. Slides were then fixed with 4% PFA, blocked with normal goat serum, stained with anti-NLRC5 primary antibody (Novus, NBP2-94,762), rinsed, stained with secondary antibody (Alexa 594 Goat anti-Rabbit, Jackson ImmunoResearch) or with anti-STAT1 PE (BD558537) and Flash Phalloidin Green (BioLegend), rinsed again, and then sealed with DAPI-containing Vectashield (VWR). Slides were imaged with a Leica SP8 confocal microscope, with identical laser settings across treatment groups.

### The cancer genome atlas (TCGA) data

TCGA was mined for data using cBioPortal (cbioportal.org, Firehouse Legacy dataset). A multigene query was performed, then mRNA data were exported, and Spearman correlation was determined.

### Statistical analyses

Data were analyzed by Student’s *t* test, one- or multi-way ANOVA with post hoc Tukey analyses where appropriate. GraphPad Prism software was used for graphing and statistical testing, with *p* < 0.05 considered statistically significant.

## Results

### Inducible APM expression is tightly linked to STAT1 expression, with p53 playing a minor role

**Fig. 2:**
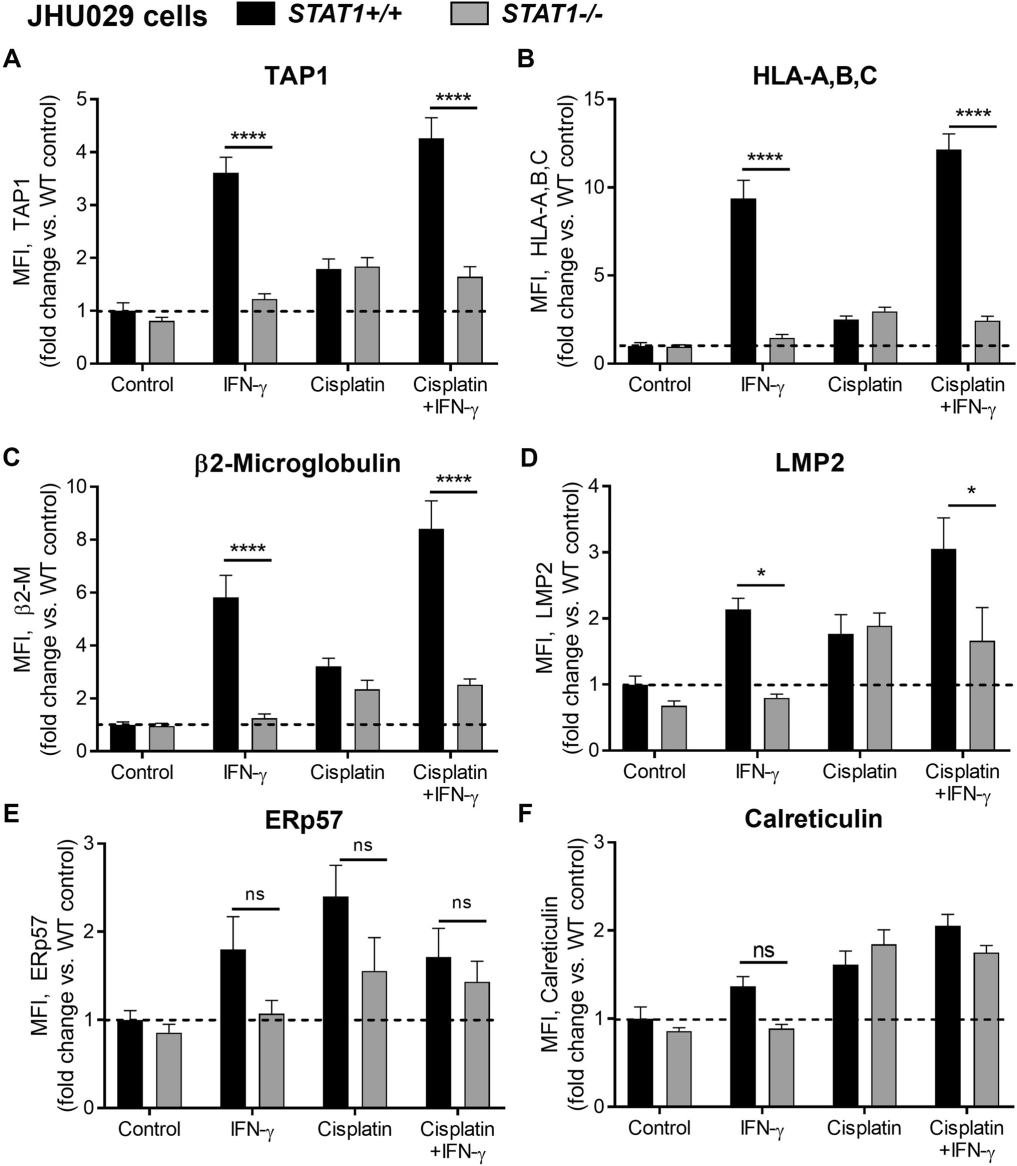
*STAT1* knockout abolishes inducible antigen processing machinery (APM) component expression after treatment with IFN-γ, but not cisplatin, in p53-null JHU029 cells. Cells were treated with IFN-γ (10 ng/ml) or a sublethal dose of cisplatin for 48 h, then fixed, stained for APM components, and analyzed by flow cytometry. *p < 0.05, ****p < 0.0001 by three-way ANOVA with post hoc Tukey comparison. In wild-type cells, IFN-γ alone induced statistically significant increases in TAP1, HLA-A,B,C, and β2-microglobulin (*p* < 0.0001), and cisplatin alone induced statistically significant increases only for ERp57 (*p* < 0.05) and calreticulin (*p* < 0.01). Results are mean + SEM, *n* = 6, combined from two independent experiments done in triplicate

To evaluate the effects of p53 and STAT1 on baseline and inducible APM expression, cell lines were treated with control media, IFN-γ, and/or cisplatin for 48 h and then evaluated for expression of intracellular APM components by flow cytometry, as previously described [10, 15]. To evaluate the significance of *TP53* loss, cisplatin was utilized for its ability to cause DNA damage and p53 activation. JHU029 cells express high STAT1 but are null for *TP53* [18] and, as expected, did not show a significant increase in APM components after a 48-h incubation in 1 µg/mL cisplatin when compared to control (Fig. 2). We then knocked out *STAT1* in JHU029 cells with CRISPR-Cas9. JHU029 cells were kept as a knockout pool, but low STAT1 expression was verified routinely by flow cytometry (Supplemental Figure S1A). Without STAT1, JHU029 cells were completely unable to upregulate APM components upon treatment with IFN-γ (Fig. 2).

**Fig. 3:**
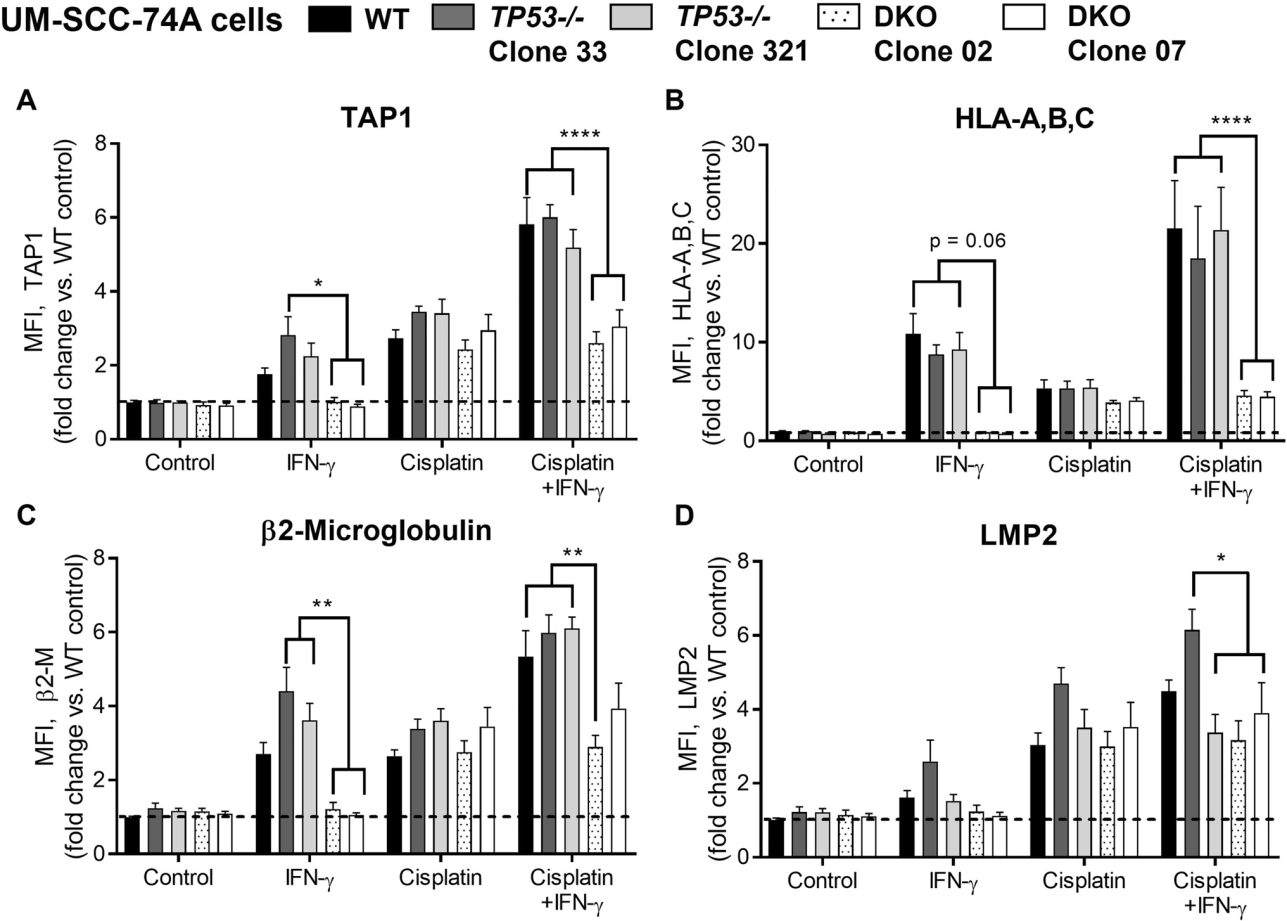
Knockout of *STAT1*, but not *TP53*, abolishes inducible APM component expression in UM-SCC-74A cells. Cells were treated with IFN-γ (10 ng/ml) or a sublethal dose of cisplatin for 48 h, then fixed, stained for APM components, and analyzed by flow cytometry. *p < 0.05, **p < 0.01, ****p < 0.0001 by three-way ANOVA with post hoc Tukey comparison. In wild-type cells, IFN-γ alone did not result in any statistically significant APM increases, but cisplatin induced a significant increase in TAP (*p* < 0.05). Results are mean + SEM, *n* = 9, combined from 3 independent experiments done in triplicate

We then subjected a cell line expressing wild-type p53 (UM-SCC-74A) to *TP53* knockout and created multiple *TP53-/-* clones. Lack of p53 was verified in these clones by transcriptional activity assay and flow cytometry (Supplemental Figure S1B). Two *TP53-/-* clones were then treated with IFN-γ and/or cisplatin. There was no consistent, statistically significant reduction in the baseline or inducible APM expression after knockout of *TP53* alone (Fig. 3). We performed similar experiments in *TP53-/-* and wild-type HCT116 colorectal cancer cells, obtaining similar results (Supplementary Figure S2, A and B). We then repeated these experiments with a HNSCC cell line positive for human papillomavirus (HPV), which is expected to express low levels of p53 as a result of degradation by HPV E6 oncoprotein. Only the HPV + cell line (UPCI SCC-90) showed a completely abolished APM response to cisplatin-derived DNA damage (Supplemental Figure S2, C and D).

Based on these data, we hypothesized that HNSCC cells can overcome loss of p53 and continue to upregulate APM with appropriate stimuli so long as they still express sufficient *STAT1*. We next knocked out *STAT1* in one of the *TP53-/-* UM-SCC-74A clones to create multiple dual-knockout (DKO) clones. This DKO created a “hard” APM deficiency, wherein the ability of cells to upregulate APM components upon treatment with IFN-γ and/or cisplatin was completely abolished (Fig. 3).

**Fig. 4:**
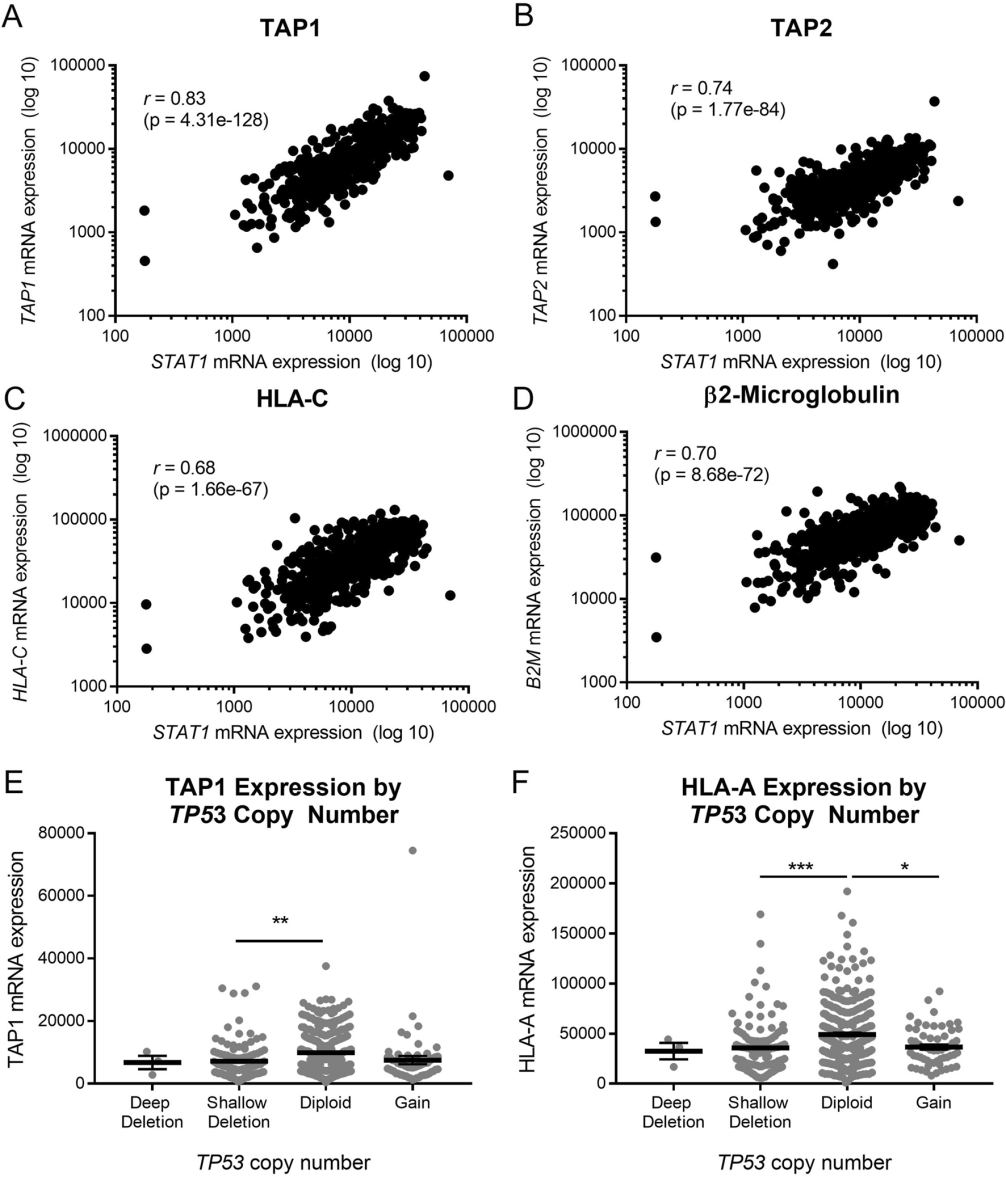
Among tumors in the Cancer Genome Atlas, APM component expression correlates strongly with expression of *STAT1* (**a**–**d**) and modestly with *TP53* copy number (**e**, **f**). *p < 0.05, **p < 0.01, ***p < 0.001 by one-way ANOVA with post hoc Tukey comparison

**Fig. 5:**
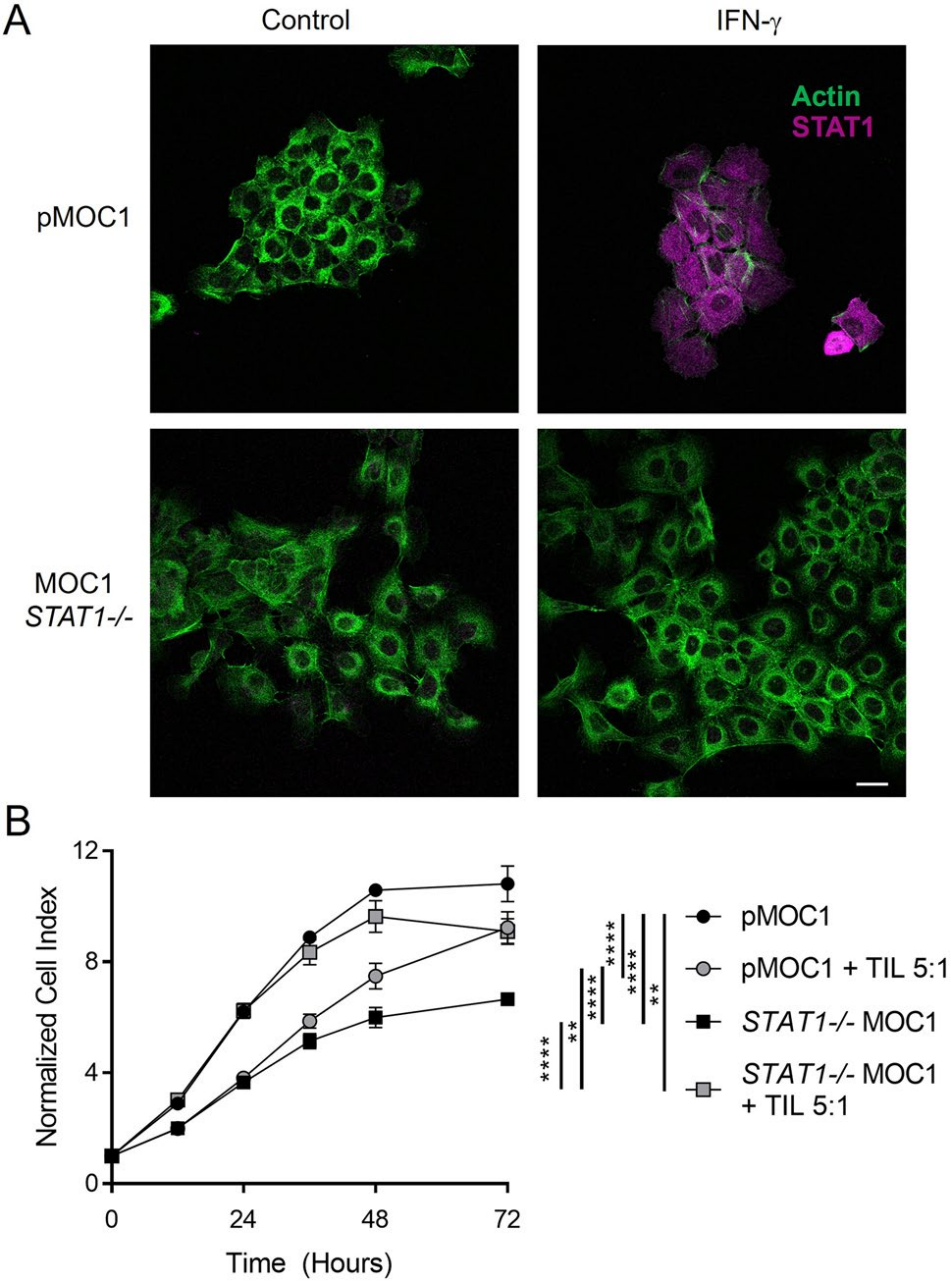
Mouse oral cancer (MOC1) cells expressing STAT1 are more efficiently killed by T cells versus *STAT1-/-* cells. In *A*, knockout of STAT1 was verified by immunofluorescence. Parental MOC1 (pMOC1) and *STAT1-/-* MOC1 cells were cultured for 24 h with control media or IFN-γ (10 ng/ml) for 24 h and then stained with phalloidin to label actin (green) and an antibody to total STAT1 (magenta). Scale bar represents 25 µm. In *B,* MOC1 cells were plated in 96-well plates and allowed to adhere overnight, then tumor infiltrating lymphocytes (TIL) were added at a 5:1 effector/target ratio. Cell growth and death were then detected as a change in electrical impedance on the xCELLigence real-time cell analyzer. Data represent mean ± SEM of 3–6 replicates, normalized to a cell index of 1.0 when TIL were added (time 0 on graph). **p < 0.01, ****p < 0.0001 by two-way ANOVA with post hoc Tukey comparison

We also examined the correlations between APM component expression and p53/STAT1 status in TCGA. As expected, we found strong correlations between mRNA levels of STAT1 and most APM components (Fig. 4a–d; Table 1). Although mRNA levels of APM components did not correlate with mRNA levels of p53 (data not shown), the mRNA levels of TAP1, HLA-A, and other APM components were found to be highest in tumors with normal diploid copy number of *TP53* (Fig. 4e–f). The end result of APM component expression and antigen processing/presentation is killing of tumor cells by T cells. To further validate the importance of STAT1 in this process, we performed T cell killing assays with mouse oral cancer (MOC1) cells lacking STAT1. Knockout of STAT1 was confirmed by immunofluorescence (Fig. 5a) and flow cytometry (Supplemental Figure S3). When tumor infiltrating lymphocytes (TIL) were added to parental MOC1 cells, a proportion of the tumor cells were killed by 72 h; in contrast, *STAT1-/-* cells were not killed when TIL were added (Fig. 5b). We actually noted that the electrical impedance increased in *STAT1-/-* cells after TIL were added, likely as a result of T cells floating to the bottom of the well without killing any tumor cells. Taken together, these data from cell lines and TCGA suggest that STAT1 is a critical transcription factor driving inducible APM component expression and ultimately T cell killing of tumor cells, but p53 does also play a minor role.

**Table 1:** Correlations of mRNA expression between STAT1/NLRC5 and antigen processing machinery (APM) components in the Cancer Genome Atlas

APM protein (Gene)	Correlation with *STAT1*: Spearman (*p* value)	Correlation with *NLRC5*: Spearman (*p* value)
TAP1	0.83 (4.31e−128)	0.80 (1.80e−111)
TAP2	0.74 (1.77e−84)	0.70 (8.93e−74)
HLA-E	0.73 (5.69e−81)	0.71 (2.25e−78)
b_2_ microglobulin	0.70 (8.68e−72)	0.66 (5.07e−64)
LMP2 (*PSMB9*)	0.70 (6.29e−74)	0.68 (3.03e−68)
LMP7 *(PSMB8)*	0.62 (2/72e−53)	0.65 (9.40e−60)
HLA-C	0.68 (1.66e−67)	0.66 (1.80e−63)
HLA-B	0.64 (2.16e−57)	0.63 (1.75e−56)
HLA-A	0.57 (1.94e−43)	0.57 (1.98e−44)
Tapasin (*TAPBP*)	0.47 (2.14e−28)	0.42 (5.02e−23)
ERAP1	0.46 (2.01e−27)	0.31 (1.69e−12)
Calreticulin	0.09 (0.0508)	0.10 (0.0227)

### PD-L1 expression reflects intact IFN/STAT1 signaling

**Fig. 6:**
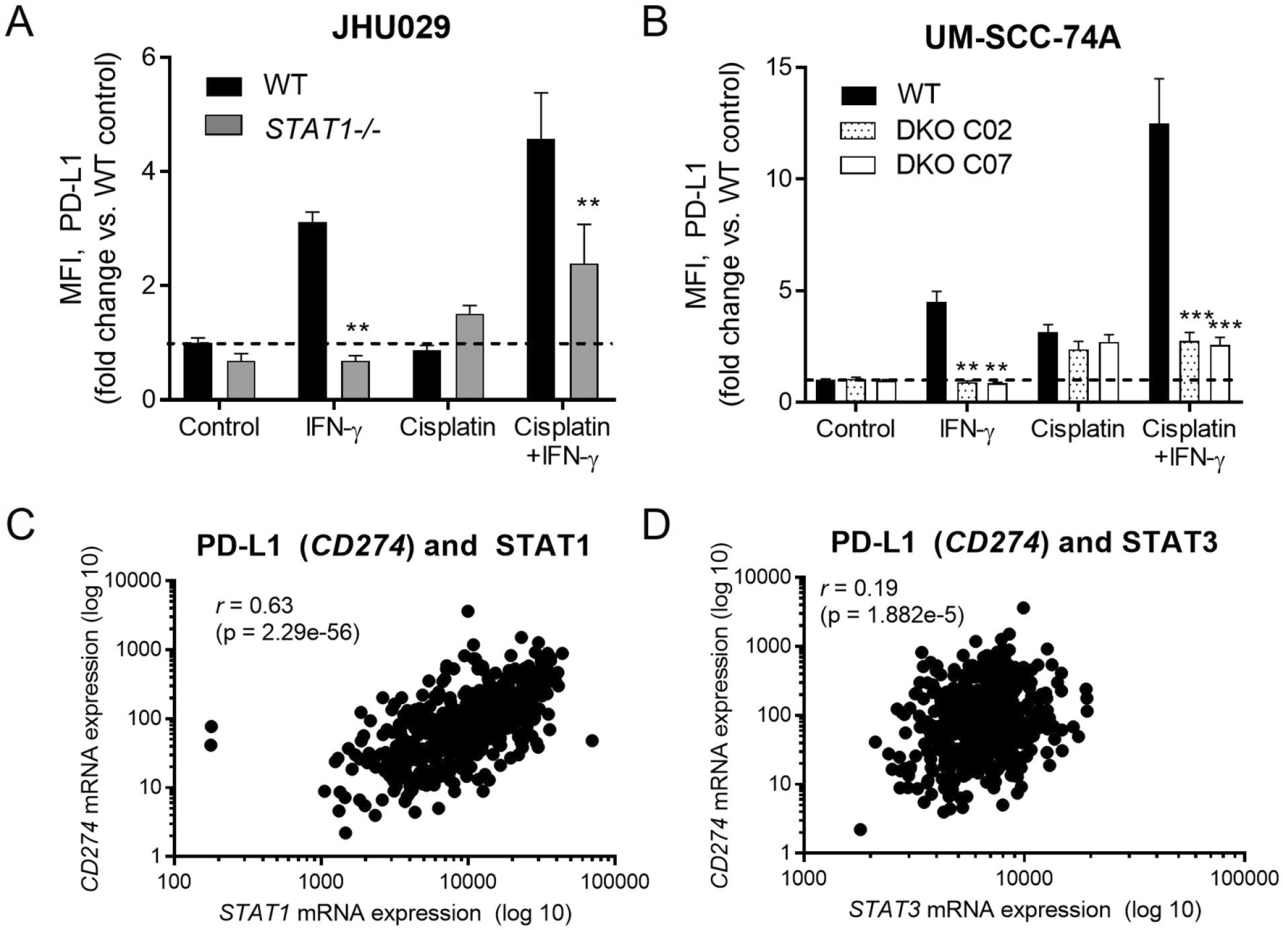
PD-L1 *(CD274)* expression correlates with expression of STAT1. In JHU029 (**a**) or UM-SCC-74A cells (**b**), knockout of *STAT1* (dual knockout of *TP53* and *STAT1* in the case of UM-SCC-74A) abolished increases in PD-L1 expression induced by IFN-γ, but not cisplatin. Cells were treated with IFN-γ (10 ng/ml) or a sublethal dose of cisplatin for 48 h, then fixed, stained for intracellular PD-L1, and analyzed by flow cytometry. **p < 0.01, ***p < 0.001 compared with wild type for same treatment group by three-way ANOVA with post hoc Tukey comparison. Results are mean + SEM, *n* = 6–9 combined from 2–3 independent experiments done in triplicate. In **c** and **d***,* expression of PD-L1 *(CD274)* correlated strongly with *STAT1* (**c**) and weakly with *STAT3* (**d**) expression in the Cancer Genome Atlas

In clinical practice for HNSCC, PD-L1 expression is the most commonly used biomarker for response to ICB. Patients with a combined positive score > 20 are much more likely to respond, versus patients with lower PD-L1 expression scores [1]. It has been suggested that these high PD-L1 expression scores are simply a reflection of strong underlying IFN signaling, since STAT1 upregulates PD-L1 in addition to APM components [19]. However, PD-L1 expression can also be driven by STAT3 [19], which functions as an oncogene and is associated with immunosuppressive myeloid cells [20–22]. To determine to what degree PD-L1 expression depends on STAT1, we examined inducible PD-L1 expression upon treatment of our cell lines with IFN-γ and/or cisplatin, which also activates STAT1 and promotes PD-L1 expression [8, 10]. As expected, the ability of HNSCC cells to increase PD-L1 expression in response to IFN-γ correlated strongly with STAT1 expression in a similar manner to the APM components (Fig. 6, A and B). In TCGA, PD-L1 expression correlated strongly with *STAT1* and weakly with *STAT3* (Fig. 6, C and D). These data suggest that high PD-L1 expression may reflect strong STAT1 signaling in HNSCC cells, which may explain why very high PD-L1 expression correlates with response to ICB.

### NLRC5 expression follows STAT1 and correlates strongly with inducible APM expression

**Fig. 7:**
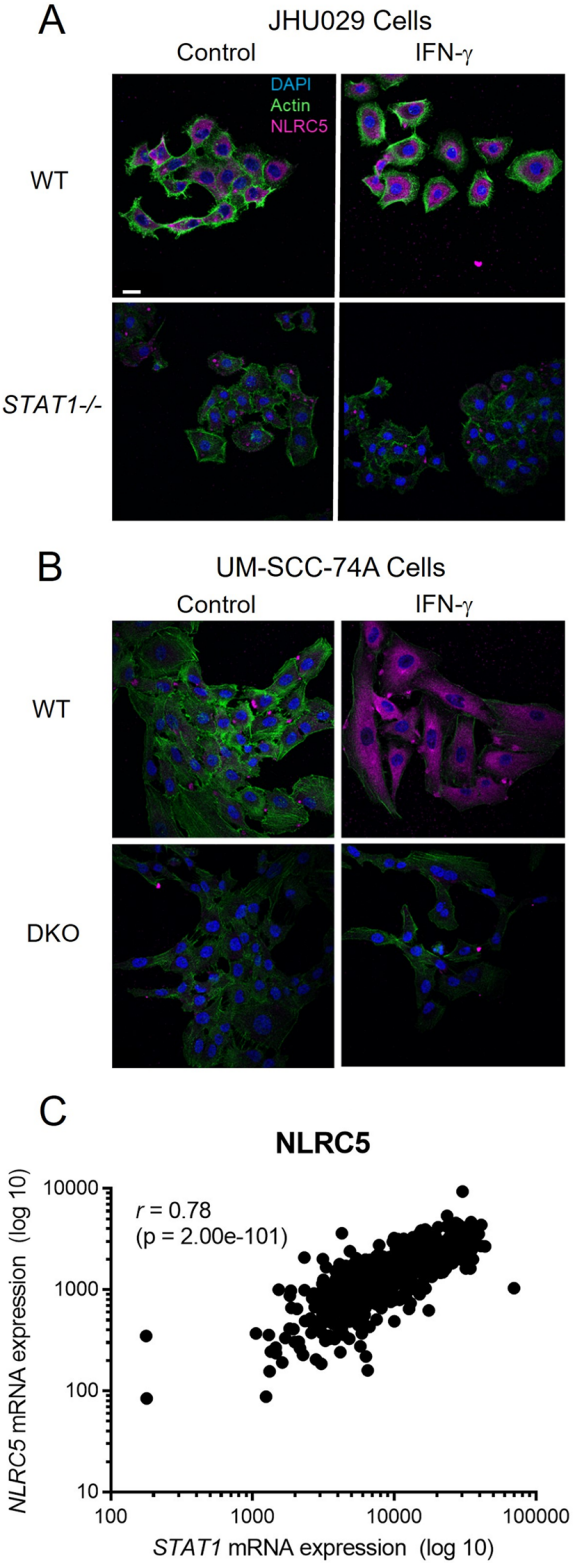
NLRC5 expression correlates strongly with expression of STAT1*.* In JHU029 (**a**) or UM-SCC-74A cells (**b**), knockout of *STAT1,* or dual knockout (DKO) of *TP53* and *STAT1* in the case of UM-SCC-74A, abolished baseline and IFN-γ-induced NLRC5 expression (magenta). Cells were treated with IFN-γ (10 ng/ml) for 48 h, then fixed, stained for intracellular NLRC5, and imaged by confocal microscopy. Scale bar = 25 µm. In **c**, expression of *NLRC5* correlated strongly with *STAT1* expression in the Cancer Genome Atlas

NLRC5, also known as MHC class I transactivator (CITA), has recently been recognized as the transcription factor downstream of STAT1 that is responsible for upregulation of MHC class I, TAP1, and other APM components [23, 24]. We next wanted to demonstrate whether NLRC5 expression is lost upon knockout of *STAT1* in our cell lines. Due to its relatively recent description, we were unable to find an antibody targeting NLRC5 that has been validated for flow cytometry. Instead, we used immunofluorescence microscopy as a semi-quantitative technique to capture changes in NLRC5 expression. It is readily visible in Fig. 7a, b that a lack of STAT1 leads to a lack of NLRC5 protein expression both at baseline and in response to IFN-γ; p53 loss did not have a significant effect on NLRC5 expression (Supplemental Figure S4). As expected, TCGA mRNA data showed a strong correlation between the expression of STAT1 and NLRC5 (Fig. 7c); consequently, high NLRC5 expression was also associated with high expression of TAP1/2, HLA, and other APM components (Table 1). Although not surprising, these data confirm that STAT1-induced NLRC5 expression is the major driver of APM component expression in HNSCC.

### Replacement of NLRC5 rescues downstream APM expression

**Fig. 8:**
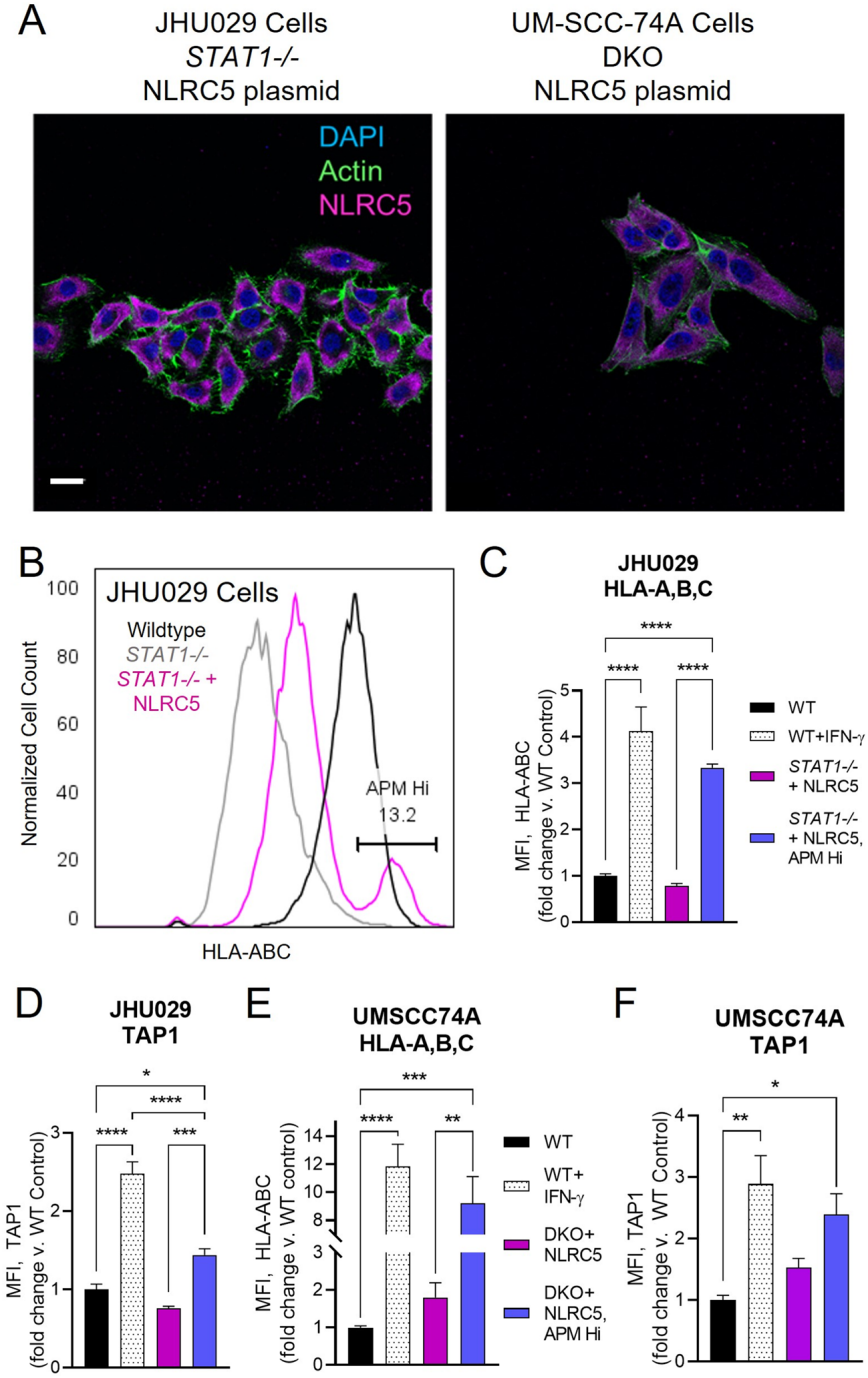
Plasmid transfection of *NLRC5* rescues APM component expression in a subset of *STAT1-/-* cells. **a** Confocal micrographs of NLRC5 expression in JHU029 *STAT1-/-* (left) and UM-SCC-74A dual-knockout cells (*TP53-/-* and *STAT1-/-,* right) following plasmid transfection and selection by G418 resistance. Cells were grown in media for 24 h, then fixed, stained for intracellular NLRC5, and imaged by confocal microscopy. Scale bar = 25 µm. **b** High levels of HLA-A,B,C expression are restored in a subset of transfected cells. Cells were grown in culture media for 24 h then fixed, stained for APM components, and analyzed by flow cytometry (JHU029 are shown). **c**–**f** Cells were grown in culture media for 24 h then fixed, stained for APM components, and analyzed by flow cytometry. *p < 0.05, **p < 0.01, ***p < 0.001, ****p < 0.0001 by two-way ANOVA with post hoc Tukey comparison. Results are mean + SEM, *n* = 6, combined from two independent experiments done in triplicate

The idea of circumventing deficient STAT1 signaling and restoring APM expression by replacing NLRC5 is highly appealing. To test this idea, we utilized a plasmid containing NLRC5 cDNA to bypass STAT1. Immunofluorescence micrographs taken after transfection and selection showed the rescue of NLRC5 protein expression (Fig. 8a). Following confirmation of NLRC5 protein expression, the transfected cells were subjected to flow cytometry to measure expression of downstream TAP1 and HLA-A,B,C compared to wild type cell lines. Transfection of NLRC5 restored high levels of HLA-A,B,C in a subset of transfected cells at baseline (Fig. 7b). When we gated on this subset of cells and compared them to the wild-type cell lines, the level of expression of both TAP1 and HLA-A,B,C was higher versus the WT control, even in the absence of stimulation with IFN-γ (Fig. 8c–f). Taken together, these results suggest that APM deficits in HNSCC can be overcome by restoring NLRC5 expression, effectively bypassing deficient IFN/STAT1 signaling.

## Discussion

Antigen processing and presentation is a key part of the anti-tumor immune response; without it, tumor cells are essentially “invisible” to cytotoxic T lymphocytes. Not surprisingly, deficits in APM component expression are relatively common in solid tumors. However, the exact mechanisms for APM deficits may be tumor specific. For example, melanomas often feature low levels of activated STAT1 and low APM expression as a result of upstream janus kinase (JAK) mutations [7]. In the case of HNSCC, high levels of EGFR activate the protein phosphatase SHP2, which dephosphorylates STAT1, thereby limiting IFN-inducible APM component expression. [3, 4]

In our prior work, we noted that cells expressing wild-type p53 expressed more TAP1 following DNA damage with cisplatin [10]. We did find studies suggesting that functional p53 is required for optimal transcription of TAP1 following treatment with DNA-damaging chemotherapeutics [12, 25, 26]; interestingly, one study showed that this process involves p53 binding to STAT1 [12]. Another study also demonstrated increased expression of multiple APM components in murine models of oral cancer after treatment with a nanoparticle carrying wild-type p53 [27]. It has long been known that *TP53* mutation, a common event in HNSCC, is associated with inferior treatment responses and prognosis [13], and this was assumed to be related to its function as a tumor suppressor. However, there is emerging evidence to suggest that p53 can also influence the tumor immune microenvironment [25]. Upon interpretation of our data from cell lines and TCGA, we were surprised to see that p53 mutation or loss can has only a minor effect on APM component expression in HNSCC cells so long as they express adequate STAT1.

A study in lung adenocarcinoma showed that patients with tumors showing mutation of *TP53*, in the absence of *EGFR* and *STK11* mutations, had higher numbers of CD8 + tumor infiltrating lymphocytes (TIL) and superior responses to PD-1 ICB; these tumors also expressed high levels of PD-L1 [28]. We posit that the high PD-L1 expression is indicative of strong underlying IFN/STAT1 signaling, and thus, the lung adenocarcinoma cells were able to produce APM components without functional p53. It is also important to note that *TP53* mutation is associated with higher tumor mutational burden (TMB). Indeed, the combination of high TMB and high IFN signaling mediators (including STAT1) is associated with excellent responses to PD-1 ICB. [29]

NLRC5/CITA is now recognized as the transcription factor downstream of STAT1 that is responsible for upregulation of the IFN-inducible APM components [24]. High levels of NRLC5 have been shown to correlate with high APM component expression, higher infiltration of CD8 + TIL, and improved survival in multiple tumor types, including HNSCC [24]. Our data suggest that the expression of NLRC5 correlates strongly with the expression of APM components, to the same degree as upstream STAT1. Consistent with this idea, we were able to increase baseline HLA and TAP1 to IFN-induced levels by rescuing expression of *NLRC5* in *STAT1-/-* HNSCC cells. Kalbasi, Ribas and colleagues previously used overexpression of *NLRC5* to rescue APM component expression and ICB sensitivity in *Jak*-knockout B16 mouse models of melanoma [6]. However, plasmid transfection in the clinical setting is challenging, so the authors explored other methods and successfully rescued APM expression with intratumoral injection of a double stranded RNA (dsRNA) analog known as BO-112 [6]. In the *Jak*-knockout B16 melanoma model, BO-112 appears to upregulate MHC class I (especially HLA-A) in a manner that is dependent on nuclear factor kappa B (NF-κB) and independent of NLRC5 [6]. It is notable that APM deficits in melanoma are often linked to *JAK* mutations, so downstream STAT1 remains intact and potentially capable of activating APM expression by other signaling pathways. In contrast, mutation or downregulation of *JAK1/2* is rare in HNSCC [30–33], but deficient expression and phosphorylation of STAT1 are common [3, 4]. Whether BO-112 or other dsRNA analogs would have similar effects on MHC class I and/or other APM components in HNSCC cells, and whether these strategies would actually restore ICB sensitivity in HNSCC, remains unclear.

One other important factor that we did not explore in depth is the relationship between p53 and APM component expression in HPV-positive versus HPV-negative HNSCC. Although *TP53* mutation is far less common in HPV-positive versus HPV-negative tumors, the E6 oncoprotein is known to degrade p53. Thus, as it pertains to APM component expression, HPV-positive tumors are subject to the same phenomenon: lack of functional p53 that may lead to a “soft” deficiency of APM component expression. In addition to this effect of E6 on p53, other HPV oncoproteins (E5, E7) can directly or indirectly reduce the expression of MHC class I and other APM components, which has been demonstrated in several prior studies [34–39]. Thus, despite high expression of IFN-γ [40], likely driven by the presence of viral material, HPV-positive tumors are also susceptible to APM deficits and resistance to immunotherapy. Based on these factors, the potential of NLRC5 rescue to mitigate APM deficits warrants further study in both HPV-positive and HPV-negative tumors.

Our study has several limitations. This work is limited to in vitro studies in cell lines, and few HNSCC cell lines with wild-type p53 are available. The lack of an NLRC5 antibody validated for quantitative techniques also required that we use a semi-quantitative technique (immunofluorescence) to assess NLRC5 protein levels. Our ongoing studies aim to see whether restoration of NLRC5 and/or use of dsRNA analog such as poly I:C can actually enhance antigen presentation, T cell-mediated tumor cell killing, and sensitivity to ICB in HNSCC models with deficient IFN/STAT1 signaling.

In conclusion, APM deficits are common in HNSCC and appear to be heavily driven by STAT1 and NLRC5, with p53 playing a minor role. Restoration or bypass of the STAT1/NLRC5 pathway may be a viable strategy for improving responses in ICB-resistant patients with HNSCC.

### Supplementary Information

Below is the link to the electronic supplementary material.

Supplementary file1 (PDF 518 kb)

## Data Availability

Data will be shared upon reasonable request.
