# Supplementary material for: Rescue of NLRC5 expression restores antigen processing machinery in head and neck cancer cells lacking functional STAT1 and p53
Source: Cancer Immunol Immunother. 2024 Jan 17;73(1):10. doi: 10.1007/s00262-023-03589-y (PMC10794329; doi:10.1007/s00262-023-03589-y)
Supplement: Supplementary file 1 — Supplementary file1 (PDF 518 kb) [file 262_2023_3589_MOESM1_ESM.pdf]

Supplementary Data

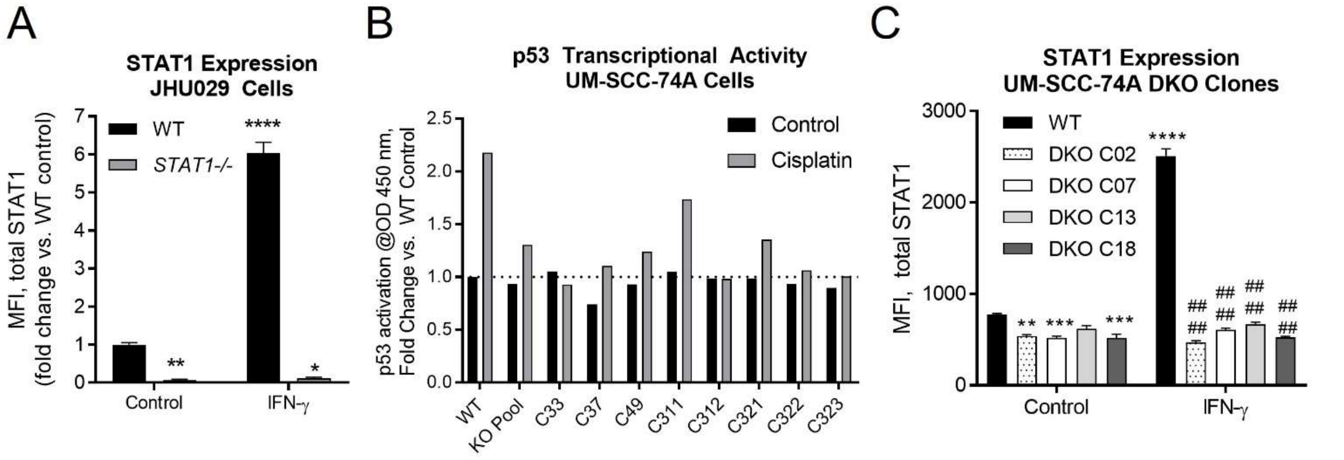

Figure S1: Verification of *TP53-/-* and *STAT1-/-* cells. (A) Wildtype (WT) JHU029 cells or cells from the *STAT1-/-* pool were treated with IFN-γ (10 ng/ml) for 48 hours, then fixed, stained for intracellular STAT1, and analyzed by flow cytometry. (B) Wildtype or TP53-/- clones of UM-SCC-74A cells were treated with control media or cisplatin (to activate p53) for 24 hours. To verify lack of the *TP53* DNA binding domain in specific clones, a transcriptional activity assay was performed on nuclear extracts for all clones. (C) Wildtype (WT) or dual knockout (DKO; *TP53-/-* and *STAT1-/-*) clones of UM-SCC-74A cells were treated with IFN-γ (10 ng/ml) for 48 hours, then fixed, stained for intracellular STAT1, and analyzed by flow cytometry. *p<0.05, **p<0.01, ***p<0.001, ****p<0.0001 versus wildtype control by two-way ANOVA with post-hoc Tukey comparison.

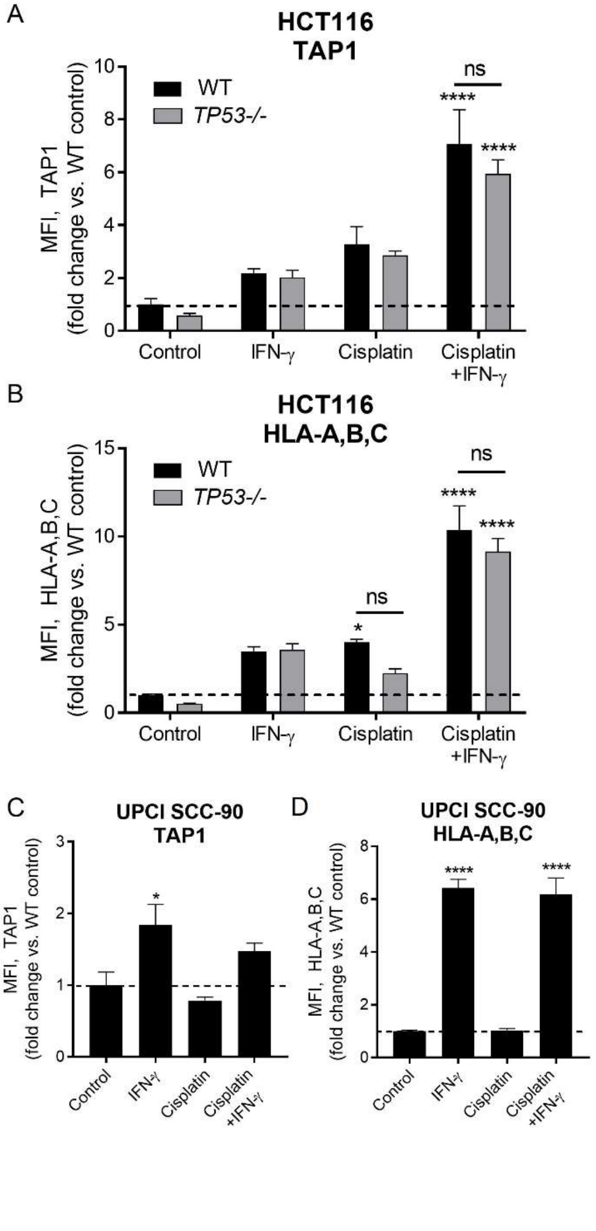

Figure S2: IFN-γ-induced TAP1 and HLA-A,B,C expression were not impaired in other cell lines lacking functional p53 either due to knockdown (HCT116 colorectal cancer cells; A,B) or degradation by HPV E6 oncoprotein (UPCI SCC-90 oropharyngeal cancer cells; C,D). Cells were treated with IFN-γ (10 ng/ml) or a sublethal dose of cisplatin for 48 hours, then fixed, stained for APM components, and analyzed by flow cytometry. *p<0.05, ****p<0.0001 versus wildtype control by three-way (A, B) or two-way (C,D) ANOVA with post hoc Tukey comparisons. Results are mean + SEM, n = 6, combined from two independent experiments.

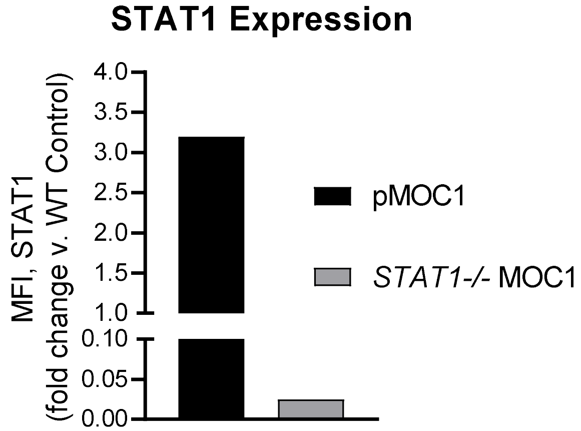

Figure S3: Knockout of STAT1 from mouse oral cancer (MOC1) cells was verified by flow cytometry. Cell lines were cultured with control media or interferon-gamma (10 ng/ml) for 24 hours, then fixed, permeabilized, and analyzed by flow cytometry, with values above normalized to wildtype control (= 1, not shown).

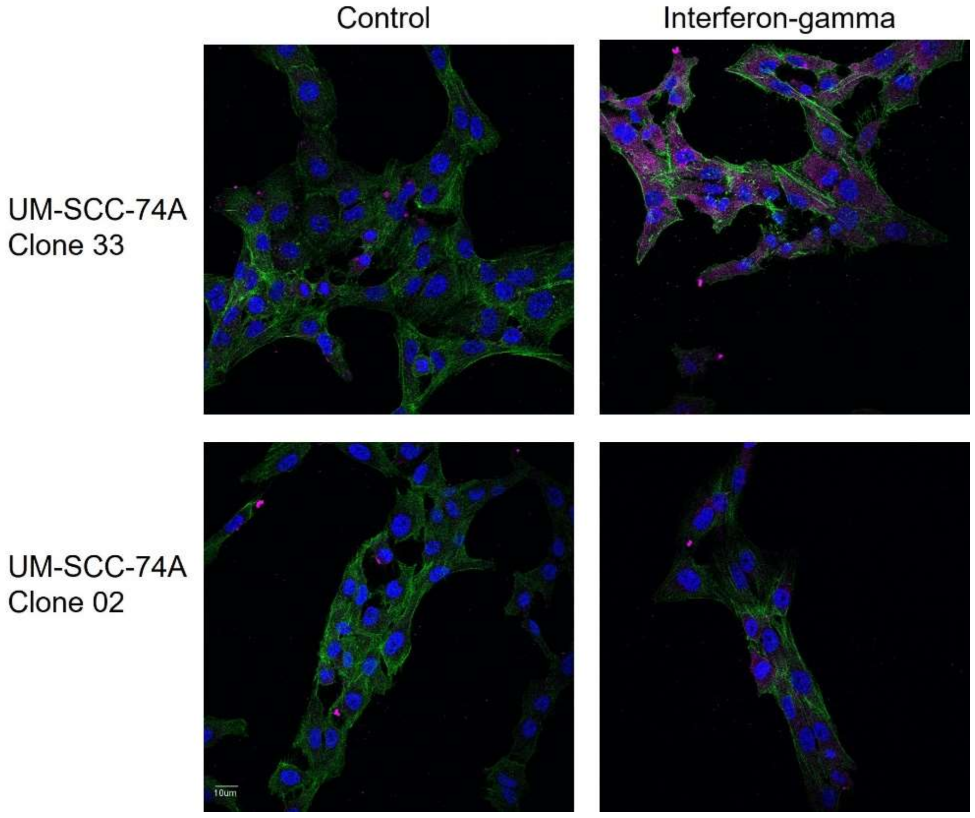

Figure S4: Confocal micrographs of NLRC5 expression in UM-SCC-74A clone 33 and UM-SCC-74A clone 02 (*TP53-/-*). Cells were treated with IFN-γ (10 ng/ml) for 48 hours, then fixed, stained for NLRC5 and imaged by confocal microscopy. DAPI is blue, actin (phalloidin stain) is green, and NLRC5 is magenta.
